# Atypical Kaposi Sarcoma in HIV Infection with Predominant Nodal Involvement

**DOI:** 10.7759/cureus.114648

**Published:** 2026-08-17

**Authors:** Maria Dioselina Ruiz Barrera, Luis Ernesto Heredia Santos, Maria Alaciel Galvan Merlos, Leticia Guerrero Navarrete, Lissette Haydee García Mena, Maria Jose Hernandez Cruz, Liliana Antonio Revuelta

**Affiliations:** 1 Adult Intensive Care Unit, Hospital Regional de Alta Especialidad de Ixtapaluca, Ixtapaluca, MEX; 2 Internal Medicine, General Regional Hospital No. 220 “General José Vicente Villada”, Instituto Mexicano del Seguro Social (IMSS), Toluca, MEX; 3 Critical Care Medicine, Hospital Regional de Alta Especialidad de Ixtapaluca, Ixtapaluca, MEX; 4 Coronary Care Unit, National Institute of Cardiology ¨Ignacio Chavez¨, Mexico City, MEX; 5 Medicine, Universidad Nacional Autónoma de México, Mexico City, MEX; 6 Medicine, Instituto Politécnico Nacional, Mexico City, MEX

**Keywords:** case report, coronavirus disease 2019 (covid-19), cutaneous horn, hiv-associated malignancies, hiv diseases, inguinal lymphadenopathy, kaposi sarcoma, kaposi sarcoma in immunocompetent patient, opportunist infections in hiv, systemic disease

## Abstract

Kaposi sarcoma (KS) is an angioproliferative neoplasm driven by human herpesvirus 8 (HHV-8), most commonly occurring in immunocompromised individuals, particularly in association with HIV infection. Although it classically presents with characteristic cutaneous or mucocutaneous lesions, nodal-predominant disease without skin involvement is uncommon and may pose a significant diagnostic challenge.

We report the case of a 26-year-old man with recently diagnosed HIV infection and no prior antiretroviral therapy (ART) who presented with a two-week history of persistent fever, constitutional symptoms, abdominal pain, and progressive intolerance to oral intake. Initial evaluation revealed hemodynamic instability, severe microcytic anemia, marked thrombocytopenia, and progressive renal dysfunction. Imaging demonstrated generalized lymphadenopathy involving multiple nodal chains, bilateral pleural effusions, hepatosplenomegaly, and free intra-abdominal fluid, raising concern for a disseminated infectious, lymphoproliferative, or neoplastic process. CD4+ T-cell count and HIV viral load were not available in the clinical records reviewed; therefore, the degree of HIV-associated immunosuppression could not be quantitatively established.

Given the persistence and progression of systemic manifestations, an excisional inguinal lymph node biopsy was performed. Histopathological examination demonstrated spindle-cell proliferation with irregular slit-like vascular spaces and erythrocyte extravasation. Immunohistochemical analysis showed nuclear positivity for HHV-8 latent nuclear antigen-1 (LNA-1), together with positivity for endothelial markers, confirming KS. No cutaneous or mucosal lesions suggestive of KS were identified during the clinical course, supporting an atypical presentation with predominant nodal involvement.

The patient received supportive inpatient management, including hemodynamic stabilization and transfusional support. Despite an initial period of partial clinical stabilization, he subsequently developed acute respiratory failure with progressive clinical deterioration and died during the index hospitalization before the planned HIV- and KS-directed therapeutic strategy could be completed.

Nodal KS without cutaneous involvement is an uncommon and potentially underrecognized presentation that may mimic HIV-associated lymphoma, disseminated opportunistic infections, and HHV-8-associated multicentric Castleman disease. This case emphasizes that KS should remain in the differential diagnosis of persistent lymphadenopathy and systemic manifestations in patients with HIV even when characteristic skin lesions are absent. Because imaging findings are nonspecific, early tissue biopsy with histopathological examination and HHV-8 immunohistochemistry is essential for definitive diagnosis.

This paper was published earlier on the Research Square preprint server on April 14, 2026 (DOI: 10.21203/rs.3.rs-9363985/v1).

## Introduction

HIV infection remains a major global health challenge despite the widespread availability of antiretroviral therapy (ART), which has substantially improved survival and transformed HIV infection into a chronic and manageable condition [1]. Nevertheless, delayed diagnosis and absence or delayed initiation of ART continue to expose some patients to opportunistic infections and HIV-associated malignancies [2]. People living with HIV have an increased risk of several malignancies compared with the general population, particularly those associated with oncogenic viruses and impaired immune surveillance [3,4].

Kaposi sarcoma (KS) is an angioproliferative neoplasm strongly associated with human herpesvirus 8 (HHV-8) infection and represents one of the classical AIDS-defining malignancies. Its development reflects the interaction between HHV-8 oncogenic activity, host immune dysfunction, chronic inflammatory signaling, and angiogenic mechanisms [5,6]. Histologically, KS is characterized by spindle-cell proliferation, irregular slit-like vascular spaces, erythrocyte extravasation, and inflammatory infiltrates. Demonstration of HHV-8 latent nuclear antigen-1 (LNA-1) by immunohistochemistry is a key diagnostic feature, particularly when the clinical presentation is atypical.

The clinical spectrum of HIV-associated KS is heterogeneous. Cutaneous lesions, typically appearing as violaceous macules, plaques, or nodules, are the most recognizable manifestations; however, mucosal, lymphatic, and visceral involvement may also occur [6,7]. Visceral disease may involve the gastrointestinal tract, lungs, liver, and lymphatic system and can present with nonspecific systemic manifestations. Consequently, the absence of characteristic skin lesions does not exclude KS and may substantially complicate clinical recognition.

Lymph node involvement can occur as part of disseminated KS, but predominant nodal disease without evident cutaneous or mucosal involvement is uncommon and represents a particularly challenging presentation. Patients may present with generalized lymphadenopathy, constitutional symptoms, cytopenias, organomegaly, or other manifestations of systemic disease. These findings overlap considerably with other conditions frequently encountered in patients with HIV, including non-Hodgkin lymphoma, tuberculosis and other disseminated opportunistic infections, systemic fungal infections, and HHV-8-associated multicentric Castleman disease [8,9]. This overlap may initially favor infectious or lymphoproliferative diagnoses and delay consideration of KS.

Imaging is valuable for identifying the distribution and extent of lymphadenopathy and visceral abnormalities but generally lacks sufficient specificity to distinguish KS from infectious or other neoplastic processes. Therefore, persistent or otherwise unexplained lymphadenopathy frequently requires tissue sampling for definitive diagnosis. Histopathological examination combined with immunohistochemical demonstration of HHV-8 is particularly important in nodal-predominant cases in which characteristic cutaneous findings are absent [6]. Recognition of this atypical phenotype is clinically relevant because available evidence remains largely derived from case reports and small series, and non-classical presentations may remain underrecognized [10,11].

We report the case of a 26-year-old man with recently diagnosed HIV infection who had not previously received ART and presented with persistent systemic symptoms, severe cytopenias, generalized lymphadenopathy, progressive organ dysfunction, and no characteristic cutaneous or mucosal lesions of KS. The diagnosis was ultimately established by lymph node histopathology and HHV-8 immunohistochemistry. This case highlights the diagnostic challenge of nodal-predominant KS and emphasizes the importance of including KS in the differential diagnosis of unexplained lymphadenopathy in patients with HIV even when its characteristic cutaneous manifestations are absent.

## Case presentation

A 26-year-old male patient, previously without known chronic medical conditions, was admitted for evaluation by the internal medicine service in the context of a febrile syndrome under investigation associated with significant hematological abnormalities. His medical history was notable for a recent diagnosis of HIV infection, confirmed a few days prior to hospital admission. At the time of initial evaluation, the patient had not initiated ART.

From a clinical perspective, the patient presented with a two-week history of progressive symptoms, including abdominal pain of nonspecific characteristics, associated with constitutional manifestations such as generalized myalgias, arthralgias, and progressive deterioration of general condition. This was followed by intolerance to oral intake, characterized by persistent nausea and vomiting of gastrointestinal content. Subsequently, the patient developed predominantly nocturnal fever accompanied by diaphoresis and subjective malaise.

From a hematological standpoint, the patient had documented severe microcytic hypochromic anemia and thrombocytopenia under investigation, suggesting the presence of an underlying systemic process. In the context of a recently diagnosed HIV infection and absence of ART, these findings raised suspicion for opportunistic infections, hematological disorders, or neoplastic processes associated with immunosuppression.

The absence of prior ART, together with the presence of systemic symptoms and cytopenias, represented a relevant clinical scenario that required a comprehensive diagnostic approach, given the increased risk of HIV-associated malignancies, including KS.

Clinical findings

At the time of admission to the Internal Medicine Service, the patient was hemodynamically stable compared to the initial evaluation in the emergency department. Vital signs were as follows: blood pressure 130/75 mmHg, heart rate 75 beats per minute, respiratory rate 20 breaths per minute, body temperature 36.5°C, and oxygen saturation 92% on room air.

On general examination, the patient was conscious, alert, and oriented, without focal neurological deficits. Mucocutaneous assessment revealed marked pallor consistent with severe anemia; however, hydration status was preserved. No cutaneous or mucosal lesions suggestive of KS, such as violaceous macules, plaques, or nodules, were identified throughout the examination.

Head and neck evaluation showed a cylindrical neck without jugular venous distention or vascular bruits. No palpable cervical lymphadenopathy was identified at that time. The oral cavity was unremarkable, with no ulcerative lesions or vascular proliferations.

Cardiopulmonary examination revealed symmetrical thoracic expansion. Pulmonary auscultation demonstrated preserved vesicular breath sounds in both hemithoraces without adventitious sounds. Cardiac auscultation revealed a regular rhythm with normal heart sounds, without murmurs or gallop.

Abdominal examination showed a soft, depressible abdomen, non-tender to superficial and deep palpation, with preserved peristalsis. No palpable visceromegaly or intra-abdominal masses were identified during the initial assessment.

Examination of the inguinal region revealed a palpable lymph node in the left inguinal area measuring approximately 3 × 3 cm, of firm consistency, mobile relative to deep planes, and non-tender on palpation. This finding was clinically significant in the context of febrile syndrome and underlying immunosuppression.

Evaluation of the extremities showed symmetric limbs without edema or peripheral vascular compromise. Distal pulses were palpable, and capillary refill time was approximately two seconds.

Overall, the combination of febrile syndrome, absence of cutaneous lesions, presence of peripheral lymphadenopathy, and systemic findings in a patient with recently diagnosed HIV infection raised suspicion for a systemic process, including opportunistic infections, lymphoproliferative disorders, or neoplasms associated with immunosuppression.

The chronological evolution of the patient’s clinical course is summarized in Table 1.

**Table 1:** Clinical timeline of the patient. The timeline summarizes the principal clinical, diagnostic, radiological, and evolutionary events from symptom onset through histopathological diagnosis and the final in-hospital outcome.

Clinical Event	Description
Two weeks prior to hospital admission	Onset of abdominal pain, arthralgias, myalgias, intolerance to oral intake, nausea, and vomiting, followed by predominantly nocturnal fever and diaphoresis.
One week prior to hospital admission	HIV infection was diagnosed. Antiretroviral therapy had not been initiated before hospitalization.
Day 1	Presentation to the emergency department because of persistent fever and deterioration in general condition. Initial vital signs were blood pressure 85/51 mmHg, heart rate 131 beats per minute, respiratory rate 23 breaths per minute, temperature 39.3 °C, and peripheral oxygen saturation (SpO₂) 95% on room air.
Day 1	Initial evaluation for SARS-CoV-2 infection was performed, with a negative rapid test.
Day 2	Admission to the internal medicine service for investigation of persistent febrile syndrome, cytopenias, and systemic manifestations of undetermined etiology.
Day 3	Initial chest computed tomography demonstrated mediastinal and bilateral axillary lymphadenopathy without focal pulmonary consolidation or significant pulmonary infiltrates.
Day 5	Laboratory evaluation demonstrated severe anemia (hemoglobin 5.9 g/dL), marked thrombocytopenia (28,000/µL), elevated inflammatory markers, and evidence of evolving renal dysfunction.
Day 6	Excisional biopsy of an inguinal lymph node was performed because of persistent generalized lymphadenopathy and concern for an infectious, lymphoproliferative, or HIV-associated neoplastic process.
Day 6	Arterial blood gas analysis demonstrated an acid-base disturbance in the setting of systemic illness and evolving renal dysfunction.
Day 9	Follow-up laboratory evaluation demonstrated persistent cytopenias and progressive deterioration in renal function.
Day 11	Severe thrombocytopenia persisted, with further progression of renal dysfunction and associated metabolic abnormalities.
Day 12	Follow-up computed tomography demonstrated multiple lymphadenopathies involving different nodal chains, bilateral pleural effusions, hepatosplenomegaly, and free intra-abdominal fluid, documenting progression of systemic involvement compared with the initial imaging study.
Subsequently	Histopathological examination of the excised lymph node demonstrated spindle-cell proliferation with irregular slit-like vascular spaces and erythrocyte extravasation. Immunohistochemical staining demonstrated human herpesvirus 8 (HHV-8) latency-associated nuclear antigen-1 (LNA-1) nuclear positivity, supporting the definitive diagnosis of nodal Kaposi sarcoma.
Subsequent hospital course	Despite supportive inpatient management and an initial period of partial clinical stabilization, the patient developed acute respiratory failure with progressive clinical deterioration.
Final outcome	The patient died during the index hospitalization before the planned HIV- and Kaposi sarcoma-directed therapeutic strategy could be completed.

Diagnostic assessment

Following admission to the internal medicine service, a comprehensive diagnostic evaluation was undertaken to determine the etiology of the febrile syndrome, cytopenias, and lymphadenopathy in the context of recently diagnosed HIV infection without prior ART.

**Figure 1:**
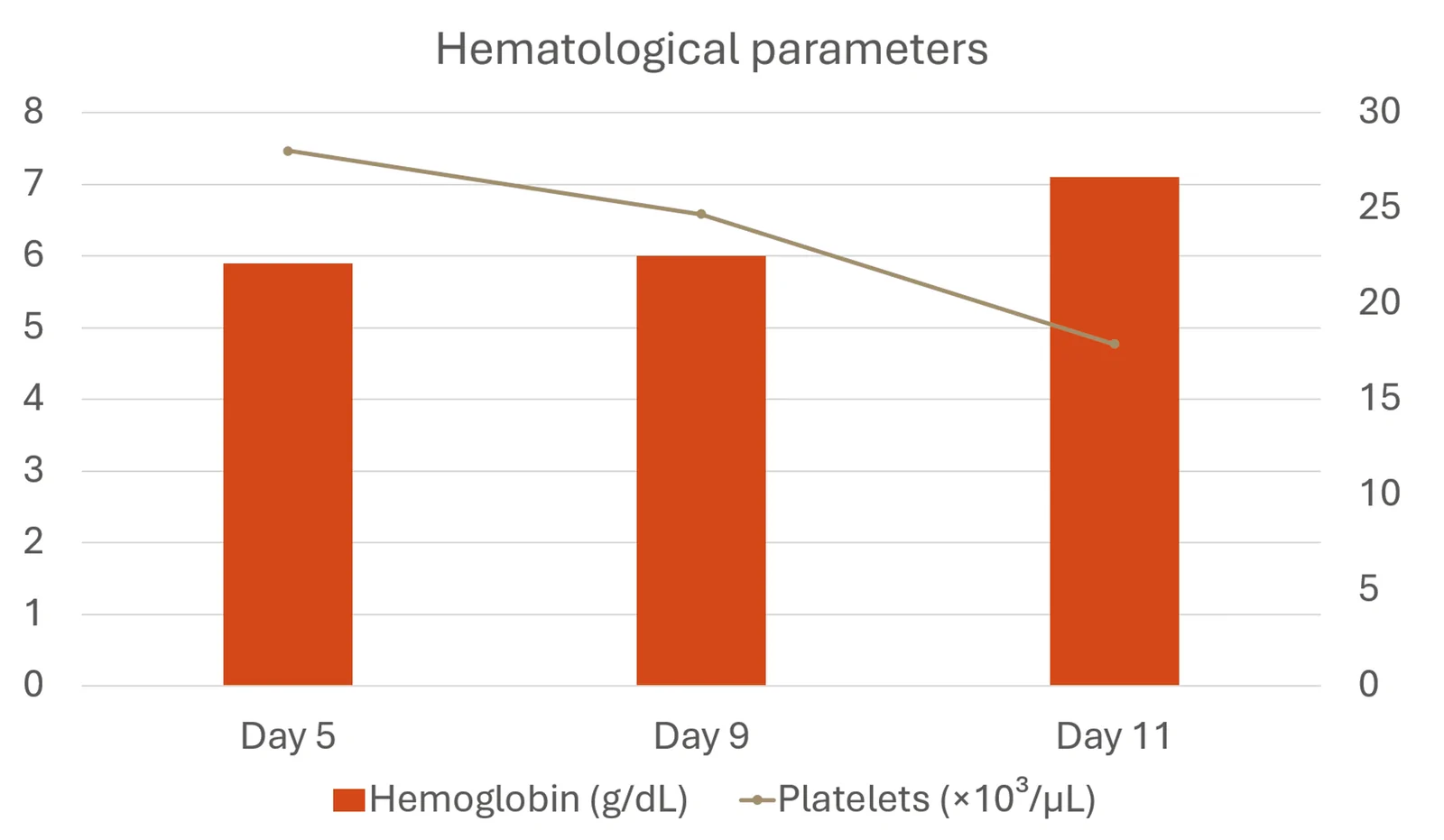
Hematological parameters during hospitalization. This figure shows the evolution of hemoglobin levels and platelet counts during hospitalization (days 5, 9, and 11). Hemoglobin values remained markedly decreased throughout the observation period, reflecting persistent severe anemia. Platelet counts demonstrated a progressive decline over time, indicating worsening thrombocytopenia during the clinical course.

**Figure 2:**
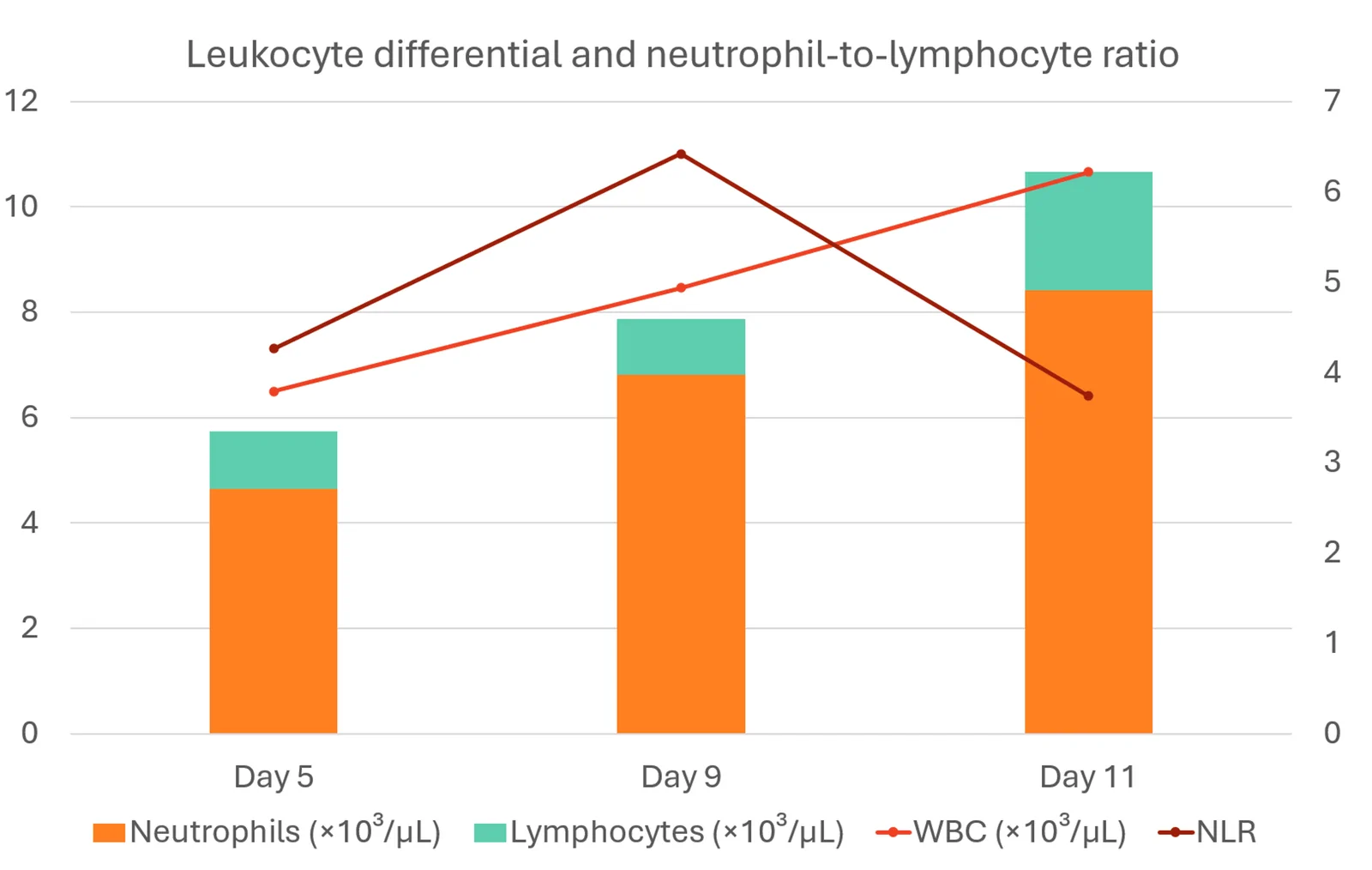
Leukocyte differential and neutrophil-to-lymphocyte ratio (NLR). This figure illustrates the trends in total leukocyte count, absolute neutrophil and lymphocyte counts, and the NLR during hospitalization. Leukocyte and neutrophil counts progressively increased, while lymphocyte levels remained relatively low. The NLR peaked around day 9, reflecting an intensified systemic inflammatory response.

Initial laboratory evaluation demonstrated severe hematological abnormalities. Hemoglobin was 5.9 g/dL with a hematocrit of 20.1%, consistent with severe anemia, while the platelet count was approximately 28,000/µL, confirming marked thrombocytopenia. The total leukocyte count was relatively preserved, with neutrophil predominance and relative lymphopenia. The temporal evolution of hemoglobin and platelet counts is presented in Figure 1, while leukocyte, neutrophil, lymphocyte, and neutrophil-to-lymphocyte ratio trends are shown in Figure 2.

Immunological staging data, including CD4+ T-cell count and plasma HIV viral load, were not available in the clinical records reviewed for this case. Therefore, the degree of HIV-associated immunosuppression could not be quantitatively established at presentation. Clinical interpretation was consequently based on the documented recent diagnosis of HIV infection, absence of prior ART, systemic manifestations, and subsequent histopathological findings.

**Figure 3:**
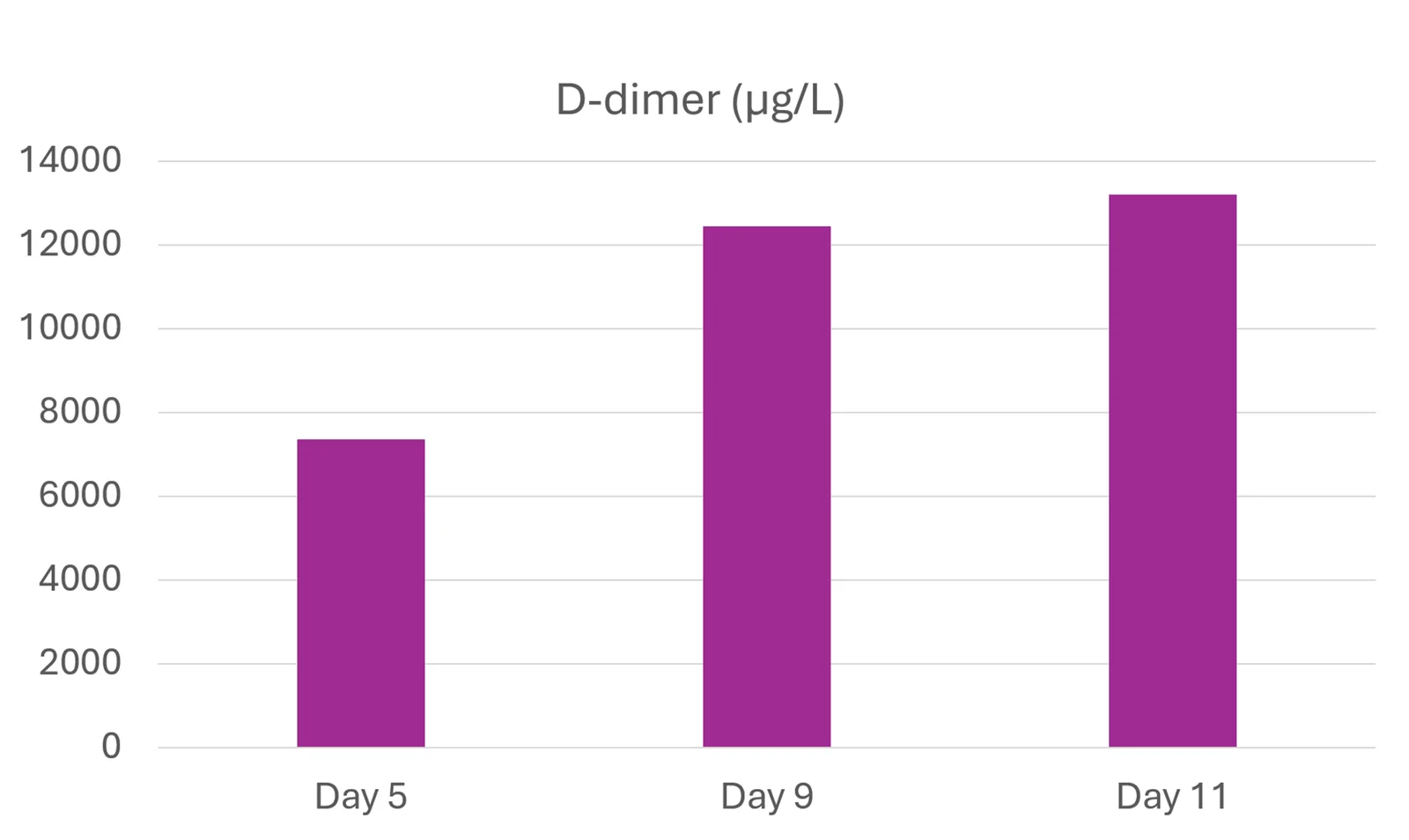
D-dimer levels during hospitalization. This figure shows the progression of D-dimer levels during hospitalization. Markedly elevated values were observed throughout the clinical course, with a progressive increase from day 5 to day 11, suggesting ongoing systemic inflammation and activation of the coagulation cascade.

Inflammatory markers were elevated, including C-reactive protein levels above 19 mg/dL. Markedly elevated D-dimer values were also documented during hospitalization. Although these findings were consistent with a systemic inflammatory and prothrombotic state, they were considered nonspecific and could not independently distinguish between infectious, inflammatory, or neoplastic etiologies. The temporal trend in D-dimer values is shown in Figure 3.

**Figure 4:**
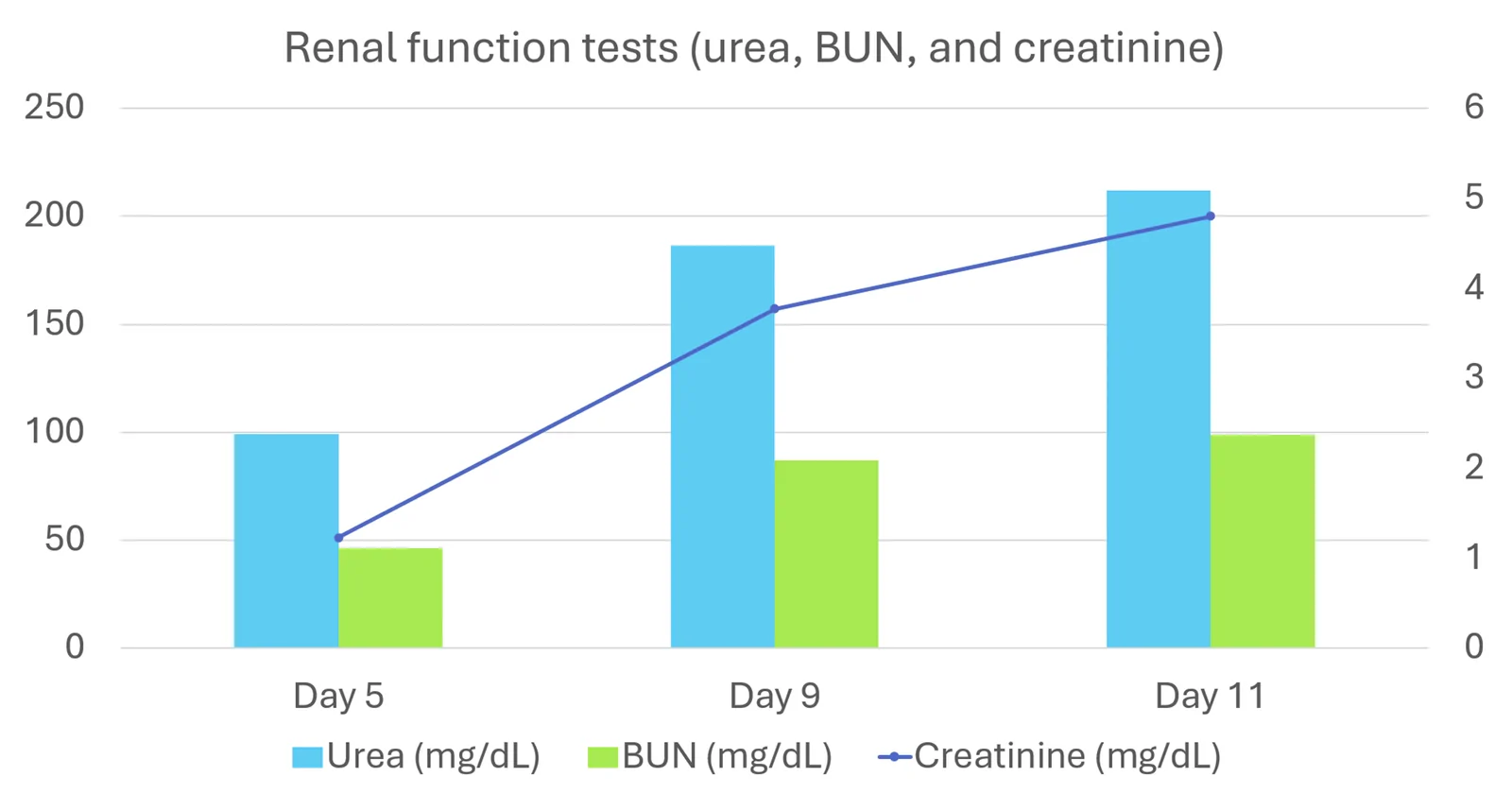
Renal function tests during hospitalization. This figure presents the temporal changes in renal function markers, including urea, blood urea nitrogen (BUN), and serum creatinine levels. All parameters showed a progressive increase from day 5 to day 11, indicating worsening renal dysfunction during hospitalization.

Biochemical evaluation demonstrated progressive deterioration in renal function. Serum creatinine increased from 1.23 mg/dL at initial evaluation to 3.77 mg/dL and subsequently to 4.80 mg/dL, accompanied by increases in urea and blood urea nitrogen. These findings were consistent with progressive acute kidney injury during hospitalization. Given the available clinical information, its etiology was considered potentially multifactorial, and the relative contribution of hemodynamic, inflammatory, and disease-related factors could not be definitively established. The temporal evolution of renal function parameters is presented in Figure 4.

**Figure 5:**
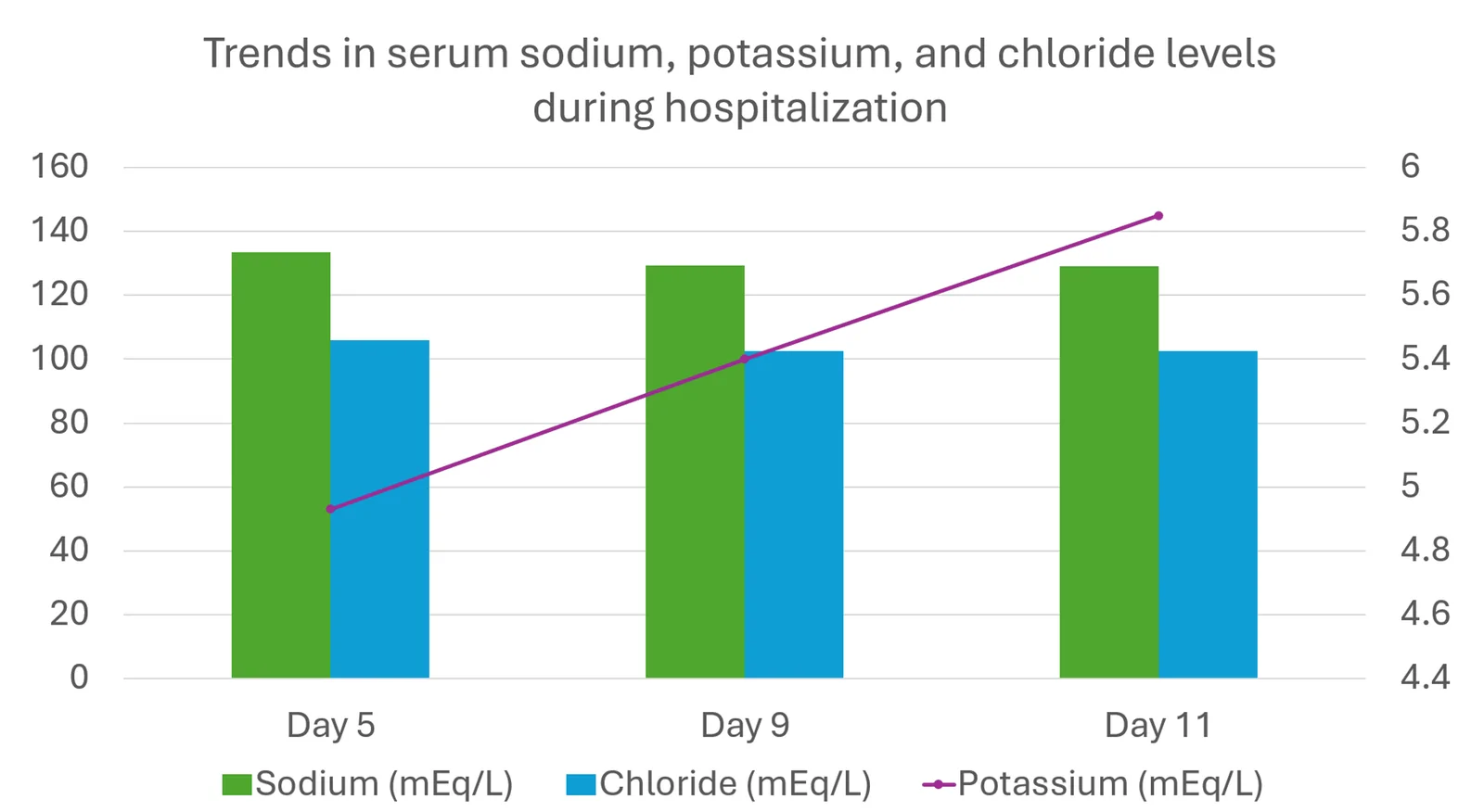
Trends in serum electrolytes during hospitalization. This figure illustrates the evolution of serum sodium, potassium, and chloride concentrations during hospitalization. Sodium and chloride levels remained relatively stable throughout the observation period, whereas potassium levels showed a gradual increase, consistent with impaired renal function.

Electrolyte abnormalities were also documented, including hyponatremia, hyperkalemia, hypocalcemia, and hyperphosphatemia. Serum albumin was markedly decreased, with values around 1.33 g/dL. These abnormalities occurred in the setting of progressive systemic illness and renal dysfunction. Trends in serum sodium, potassium, and chloride are shown in Figure 5.

Liver biochemical tests showed only mild abnormalities, with transaminase levels remaining near the reference range and minimal elevation of bilirubin. Tumor markers, including alpha-fetoprotein, carbohydrate antigen 19-9, and carcinoembryonic antigen, were within their respective reference ranges. These results did not provide evidence supporting a primary gastrointestinal or hepatobiliary malignancy.

An arterial blood gas analysis obtained during hospitalization showed a pH of 7.45 with decreased bicarbonate and a negative base excess. These findings indicated an acid-base disturbance occurring in the setting of progressive systemic illness and renal dysfunction.

**Figure 6:**
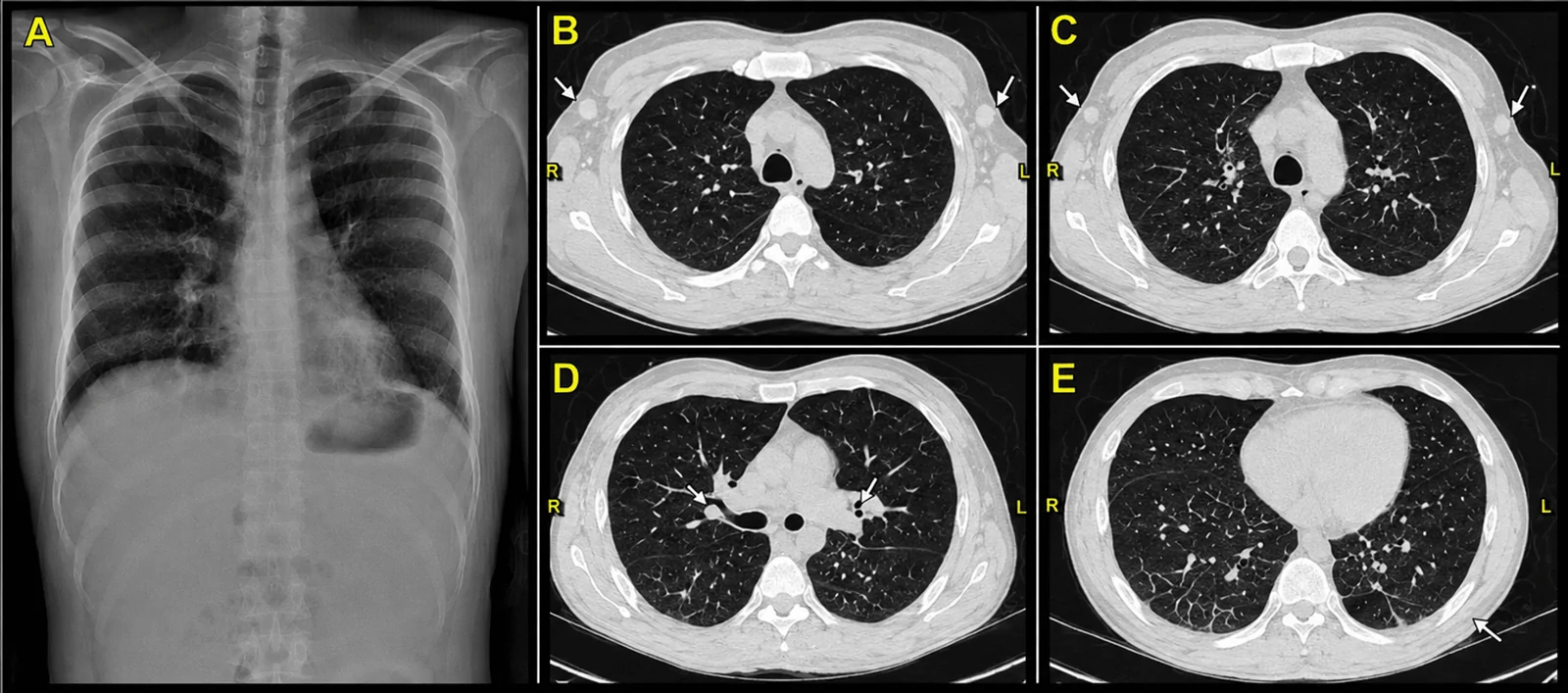
Chest imaging findings during hospitalization. Panel A:  Frontal chest radiograph showing preserved cardiomediastinal silhouette and bilateral perihilar interstitial markings without evidence of focal consolidation. No pleural effusion or pneumothorax is identified. Panels B–E. Chest computed tomography (CT) scans (axial lung window) demonstrate preserved pulmonary architecture without evidence of focal consolidation or radiological findings suggestive of acute infectious processes. A mild left basal laminar atelectasis is observed. Additionally, bilateral axillary and mediastinal lymphadenopathy are identified, without evidence of mediastinal space-occupying lesions. Overall, these findings are consistent with the absence of acute pulmonary infection, with associated lymphadenopathy suggestive of an underlying systemic or neoplastic process, as described in the clinical context.

In parallel with the laboratory evaluation, imaging studies were obtained to identify potential infectious or neoplastic processes. Initial chest computed tomography demonstrated bilateral axillary and mediastinal lymphadenopathy without focal pulmonary consolidation suggestive of an acute infectious process. Pulmonary architecture was otherwise relatively preserved, with mild left basal laminar atelectasis and no mediastinal space-occupying lesion. These findings are shown in Figure 6.

The identification of lymphadenopathy involving multiple nodal territories, including mediastinal, axillary, and inguinal regions, broadened the differential diagnosis to include lymphoproliferative disorders, disseminated infectious processes, and HIV-associated neoplasms. Because the imaging findings were nonspecific, histopathological evaluation of the affected lymph node was considered necessary to establish a definitive diagnosis.

Differential diagnosis

The initial differential diagnosis included HIV-associated non-Hodgkin lymphoma, disseminated opportunistic infections such as tuberculosis or systemic mycoses, HHV-8-associated multicentric Castleman disease, and KS with atypical nodal presentation. The absence of cutaneous lesions and the presence of generalized lymphadenopathy, cytopenias, and systemic inflammatory response initially favored infectious and lymphoproliferative etiologies over KS.

Given these findings, histopathological study of the affected lymph node through lymph node biopsy was considered essential in order to establish a definitive diagnosis and guide subsequent therapeutic management.

**Figure 7:**
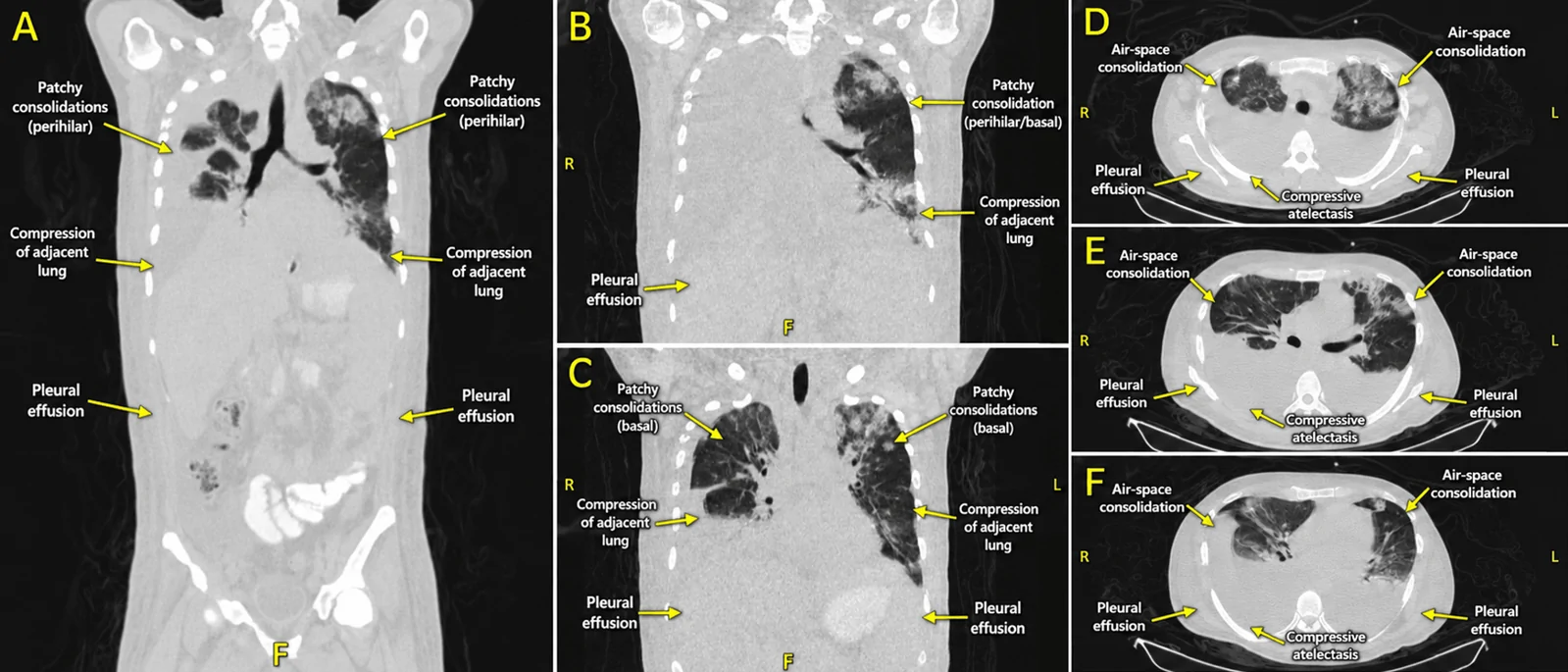
Follow-up computed tomography showing progression of thoracic involvement. Panels A–C: Coronal CT images demonstrate extensive bilateral pulmonary involvement characterized by patchy consolidations with a predominantly perihilar and basal distribution. There is evidence of associated bilateral pleural effusion occupying a significant portion of the thoracic cavity, contributing to partial compression of the underlying lung parenchyma. Additionally, findings suggest increased soft tissue density consistent with generalized lymphadenopathy and possible associated systemic involvement. Panels D–F: Axial CT images (lung window) confirm the presence of bilateral pleural effusions with compressive atelectasis of adjacent lung segments. Patchy areas of consolidation and interstitial involvement are observed, without well-defined cavitary lesions. The distribution and extent of findings are suggestive of a progressive systemic process rather than a localized pulmonary infection. Overall, the imaging findings demonstrate clear radiological progression compared to prior studies, with development of pleural effusions, worsening parenchymal involvement, and features consistent with systemic disease, correlating with the patient’s clinical deterioration.

In parallel with clinical and laboratory evaluation, imaging studies were performed with the aim of identifying possible infectious foci or neoplastic processes. Initial chest computed tomography demonstrated the presence of bilateral axillary and mediastinal lymphadenopathy, without pulmonary infiltrates suggestive of acute infectious processes. The lungs showed preserved architecture, with only mild left basal laminar atelectasis, without mediastinal space-occupying lesions. Subsequently, a follow-up chest computed tomography was performed, in which progression of findings was documented, with an increase in the number and size of lymphadenopathies in multiple nodal territories, as well as the appearance of bilateral pleural effusion, hepatosplenomegaly, and free fluid in the abdominal cavity, which oriented toward a systemic process of infiltrative or neoplastic nature as described in Figure 7.

**Figure 8:**
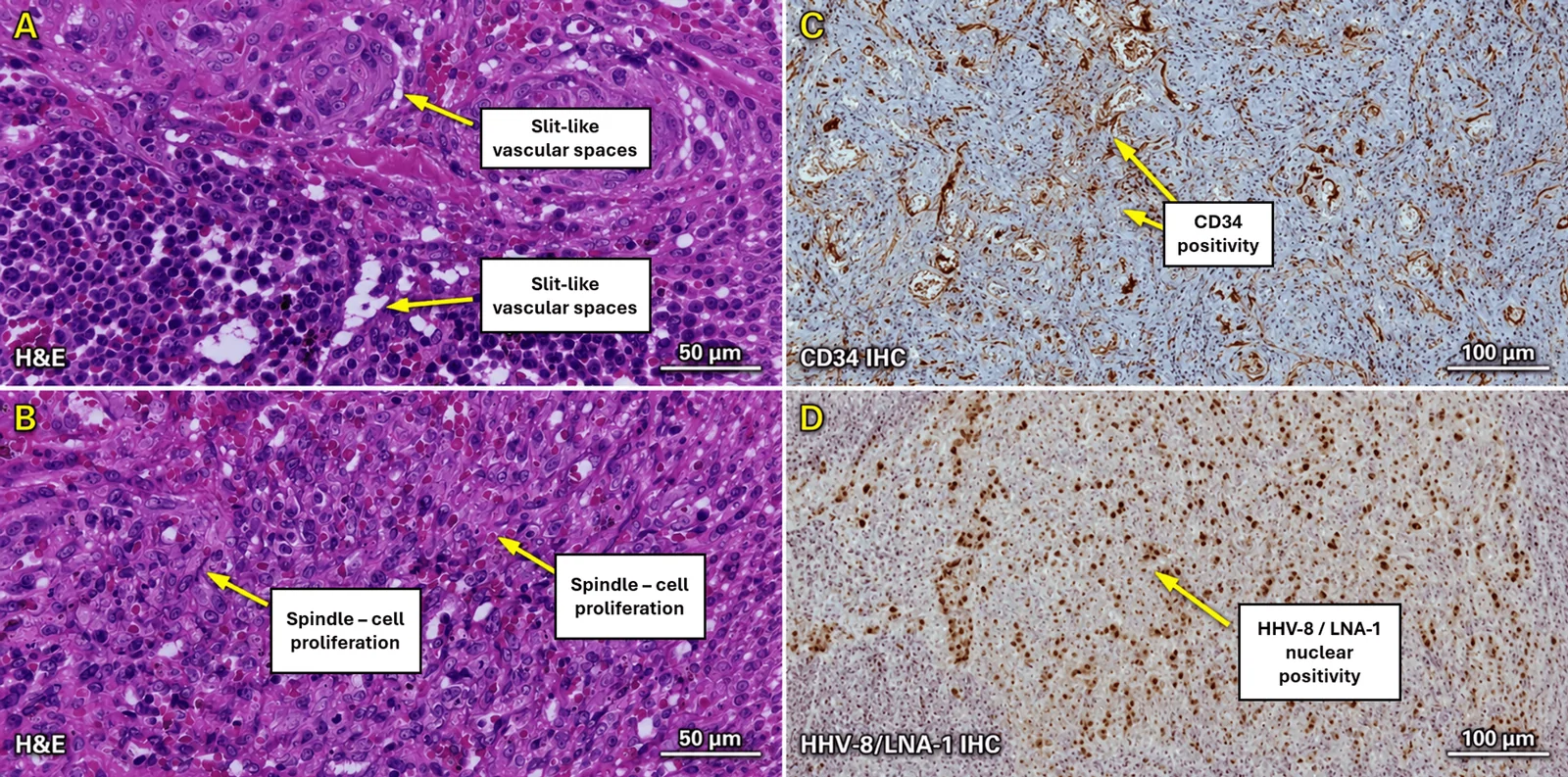
Histopathological and immunohistochemical findings of nodal Kaposi sarcoma. Panels A–B: Hematoxylin and eosin (H&E) staining; microscopic examination shows a mesenchymal vascular neoplasm characterized by a proliferation of spindle-shaped cells arranged in interlacing fascicles. Irregular, slit-like vascular spaces are observed, containing extravasated erythrocytes. The background demonstrates a mixed inflammatory infiltrate. These findings are consistent with Kaposi sarcoma. Panel C: CD34 immunohistochemistry; immunostaining reveals cytoplasmic positivity in spindle cells as well as in endothelial cells lining vascular channels, supporting the endothelial origin of the neoplastic proliferation. Panel D: Human herpesvirus 8 (HHV-8) latent nuclear antigen-1 (LNA-1) immunohistochemistry; nuclear positivity for HHV-8 LNA-1 is demonstrated, confirming the diagnosis of Kaposi sarcoma.

The presence of severe cytopenias, particularly anemia and thrombocytopenia, contributed to increasing the initial suspicion of malignant or infiltrative hematological processes. Likewise, the identification of lymphadenopathy in multiple nodal territories on imaging studies reinforced the diagnostic possibility of a lymphoproliferative disease. However, histopathological study of the lymph node allowed identification of a vascular neoplasm compatible with KS, as shown in Figure 8, and was subsequently confirmed by immunohistochemistry with positivity for HHV-8, as shown in Table 2.

**Table 2:** Immunohistochemical findings. Immunohistochemical analysis demonstrated nuclear positivity for HHV-8 (latent nuclear antigen-1 (LNA-1)), confirming the diagnosis of Kaposi sarcoma. CD34 and podoplanin (D2-40) positivity support the endothelial origin of the spindle cell proliferation.

Antibody	Clone	Staining pattern	Interpretation
Human herpesvirus 8 (HHV-8)	13B10	Nuclear	Positive
CD34	QBEND10	Cytoplasmic and membranous	Positive
Podoplanin (D2-40)	D2-40	Cytoplasmic and membranous	Positive

Diagnostic challenges

One of the main diagnostic challenges in this case was the absence of characteristic cutaneous or mucosal lesions, which are typically considered hallmark features of KS. This atypical presentation significantly reduced the initial clinical suspicion of this entity and contributed to prioritizing alternative diagnoses such as lymphoma or disseminated infection.

Additionally, the coexistence of generalized lymphadenopathy, severe cytopenias, systemic inflammatory response, and progressive organ dysfunction created substantial overlap with lymphoproliferative disorders and severe infectious processes commonly observed in patients with advanced HIV infection. Imaging findings lacked specificity and did not allow clear differentiation between infectious and neoplastic etiologies.

Consequently, definitive diagnosis relied on histopathological confirmation through lymph node biopsy, highlighting the importance of early tissue sampling in similar clinical scenarios to avoid diagnostic delay.

Prognosis

The presence of generalized nodal involvement, bilateral pleural effusions, hepatosplenomegaly, severe cytopenias, progressive renal dysfunction, and subsequent respiratory deterioration indicated a severe systemic presentation with an unfavorable in-hospital course. However, because CD4+ T-cell count and HIV viral load were not available, the degree of HIV-associated immunosuppression could not be quantitatively established or directly correlated with disease severity.

The prognosis was further limited by rapid clinical deterioration and the development of acute respiratory failure during the index hospitalization. Although ART and oncologic management are important components of treatment for HIV-associated KS, their potential impact could not be evaluated in this case because the patient died before the planned definitive therapeutic strategy could be completed.

Therapeutic intervention

The patient received supportive inpatient management directed toward stabilization of systemic and hematological abnormalities. Initial treatment included hemodynamic support, intravenous fluid therapy, close clinical monitoring, and transfusional support for severe anemia. Thrombocytopenia was monitored closely, with supportive measures implemented to reduce bleeding risk. Progressive renal dysfunction and electrolyte abnormalities were managed conservatively with optimization of fluid balance, correction of metabolic disturbances, and avoidance of potentially nephrotoxic agents.

Following histopathological and immunohistochemical confirmation of nodal KS, definitive HIV- and KS-directed treatment was planned. However, the patient subsequently developed acute respiratory failure with progressive clinical deterioration and died during the index hospitalization before the planned ART and oncologic therapeutic strategy could be completed. Therefore, no treatment-response assessment for ART or systemic oncologic therapy was possible.

Follow-up and outcomes

Despite an initial period of partial hemodynamic stabilization during hospitalization, the patient continued to exhibit severe hematological abnormalities and progressive renal dysfunction. Histopathological and immunohistochemical examination of the excised lymph node subsequently confirmed HHV-8-positive nodal KS.

During the subsequent hospital course, the patient developed progressive clinical deterioration complicated by acute respiratory failure. Despite supportive management, his condition worsened, and he died during the index hospitalization. Consequently, outpatient follow-up, definitive long-term oncologic management, and assessment of therapeutic response were not possible.

## Discussion

The present case illustrates an uncommon presentation of KS associated with HIV infection, characterized by predominant nodal involvement in the absence of cutaneous or mucosal lesions. Although KS most commonly presents with violaceous cutaneous lesions, its clinical spectrum also includes mucosal, visceral, and lymphatic involvement [6,12]. In this patient, the absence of characteristic skin findings substantially complicated the initial diagnostic approach.

The pathogenesis of HIV-associated KS is closely related to infection with HHV-8 and HIV-associated immune dysfunction. HHV-8 promotes proliferation of infected endothelial cells and angiogenesis, while impaired immune control may facilitate disease progression [4]. However, in the present case, CD4+ T-cell count and plasma HIV viral load were not available; therefore, the degree of HIV-associated immunosuppression could not be quantitatively established or directly correlated with the severity of the clinical presentation.

Predominant nodal involvement is an uncommon manifestation of KS and represents an important diagnostic challenge. Lymph node disease more frequently occurs as part of disseminated involvement, whereas nodal-predominant presentations without cutaneous lesions are less commonly reported [7,12]. In such cases, the clinical picture may closely resemble other conditions frequently encountered in patients with HIV, particularly non-Hodgkin lymphoma, disseminated opportunistic infections, systemic mycoses, tuberculosis, and HHV-8-associated multicentric Castleman disease.

In this patient, generalized lymphadenopathy, persistent fever, severe cytopenias, progressive renal dysfunction, and subsequent systemic deterioration initially supported a broad differential diagnosis that included infectious and lymphoproliferative processes. The absence of typical cutaneous KS lesions further decreased the initial clinical suspicion for this diagnosis. This illustrates the difficulty of recognizing nodal KS when its clinical presentation overlaps with other HIV-associated diseases.

Imaging studies were useful for identifying the extent and progression of systemic involvement but were not sufficient to establish the etiology. Initial computed tomography demonstrated mediastinal and bilateral axillary lymphadenopathy, whereas subsequent imaging showed more extensive lymphadenopathy, bilateral pleural effusions, hepatosplenomegaly, and free intra-abdominal fluid. These findings remained nonspecific and could occur in both infectious and neoplastic processes, reinforcing the importance of tissue diagnosis in patients with persistent or unexplained lymphadenopathy [6,7].

Definitive diagnosis was established by excisional lymph node biopsy. Histopathological examination demonstrated spindle-cell proliferation, irregular slit-like vascular spaces, and erythrocyte extravasation, while immunohistochemistry demonstrated HHV-8 LNA-1 nuclear positivity together with endothelial marker expression. These findings confirmed nodal KS and illustrate the central role of histopathological and immunohistochemical evaluation when the clinical and radiological findings are inconclusive.

The clinical course was unfavorable. Despite supportive treatment and an initial period of partial stabilization, the patient developed progressive deterioration complicated by acute respiratory failure and died during the index hospitalization. Because definitive HIV- and KS-directed therapy could not be completed, the potential effect of ART or systemic oncologic treatment cannot be assessed from this case. Although ART remains a fundamental component of the management of HIV-associated KS and systemic therapy may be indicated in selected patients with extensive disease, this report does not permit conclusions regarding treatment efficacy or its potential impact on the outcome of this individual patient [4].

This case highlights the need to consider KS in the differential diagnosis of patients with HIV who present with persistent generalized lymphadenopathy and systemic manifestations even when characteristic cutaneous or mucosal lesions are absent. Early tissue sampling may be particularly valuable when imaging findings are nonspecific and infectious or lymphoproliferative disorders remain competing diagnostic considerations. Increased awareness of nodal-predominant KS may facilitate recognition of this uncommon clinical phenotype and support more timely diagnostic evaluation.

Limitations

This case has several limitations. First, long-term follow-up and assessment of treatment response were not possible because the patient developed acute respiratory failure and died during the index hospitalization before completion of the planned HIV- and KS-directed therapeutic strategy. Second, immunological staging parameters, including CD4+ T-cell count and plasma HIV viral load, were not available in the clinical records reviewed for this case. Consequently, the degree of HIV-associated immunosuppression could not be quantitatively established, and no direct relationship between immunological status and disease severity can be inferred. Finally, as a single case report, these observations cannot be generalized; however, they may contribute to increasing awareness of atypical nodal presentations of KS in patients with HIV.

Clinical implications

Despite these limitations, this case emphasizes the importance of maintaining a high index of suspicion for KS in patients with HIV who present with unexplained lymphadenopathy and systemic manifestations, even in the absence of cutaneous lesions. Early consideration of KS within the differential diagnosis and prompt histopathological evaluation are essential to avoid diagnostic delays and to initiate timely and appropriate management.

## Conclusions

KS associated with HIV infection remains an important diagnostic consideration, particularly in patients with recently diagnosed or untreated HIV infection. Predominant nodal involvement without cutaneous or mucosal lesions is uncommon and may closely mimic HIV-associated lymphoma, disseminated opportunistic infections, or HHV-8-associated multicentric Castleman disease. Therefore, persistent lymphadenopathy accompanied by systemic manifestations in a patient with HIV should prompt consideration of KS even when the characteristic cutaneous findings are absent.

A comprehensive diagnostic approach integrating clinical findings, laboratory abnormalities, imaging, and timely tissue biopsy is essential because radiological findings are nonspecific. Histopathological examination with immunohistochemical demonstration of HHV-8 remains fundamental for definitive diagnosis. In this case, rapid clinical deterioration and in-hospital death prevented assessment of the potential impact of definitive HIV- and KS-directed therapy. This report broadens awareness of atypical nodal presentations of KS and emphasizes the importance of early histopathological confirmation in patients with unexplained lymphadenopathy.
